# Consensus on Symptom Selection for Endometriosis Questionnaires: A Modified e‐Delphi Study

**DOI:** 10.1111/1471-0528.18066

**Published:** 2025-01-13

**Authors:** Tong Zhu, Henrik Marschall, Karina E. Hansen, Andrew W. Horne, Lucky Saraswat, Krina T. Zondervan, Stacey A. Missmer, Lone Hummelshoj, Atilla Bokor, Camilla S. Østrup, Anna Melgaard, Dorte Rytter

**Affiliations:** ^1^ Department of Public Health Aarhus University Aarhus Denmark; ^2^ Centre for Reproductive Health Institute of Regeneration and Repair, University of Edinburgh Edinburgh UK; ^3^ Aberdeen Royal Infirmary University of Aberdeen Aberdeen UK; ^4^ Oxford Endometriosis CaRe Centre, Nuffield Department of Women's and Reproductive Health University of Oxford Oxford UK; ^5^ Department of Obstetrics Gynecology and Reproductive Biology Michigan State University East Lansing Michigan USA; ^6^ Endometriosis.org London UK; ^7^ Department of Obstetrics and Gynecology Semmelweis University Budapest Hungary

**Keywords:** Delphi study, early detection, endometriosis, infertility, menstrual pain, pelvic pain, symptom assessment

## Abstract

**Objective:**

To build consensus on most important symptoms and related consequences for use in questionnaires to characterise individuals with suspected and confirmed endometriosis in the general population.

**Design:**

A questionnaire of 107 symptoms and related consequences of endometriosis was collaboratively developed by patients, medical doctors and researchers and further assessed in a two‐round e‐Delphi study. Participants assessed the relevance of the symptoms, and a priori it was decided that 70% was the threshold for inclusion of a symptom.

**Setting:**

Participants represented 7 countries, including Australia, Denmark, France, Hungary, the United Kingdom, the United States, and Turkey.

**Population:**

Individuals with endometriosis, medical doctors and researchers with expertise in endometriosis.

**Methods:**

A modified e‐Delphi study.

**Main Outcome Measures:**

Consensus‐based selection of symptoms for endometriosis questionnaires.

**Results:**

Seventy‐six participants completed the first Delphi round and 65 completed the second round. Four symptoms met consensus in the first round (menstrual pain, pain during sexual intercourse, cyclic pain during defecation, and infertility), with two additional symptoms reaching consensus in the second round (cyclic pain and increased doctor/health care contacts for abdominal/pelvic pain).

**Conclusion:**

This study highlighted six symptoms relevant for inclusion in endometriosis research questionnaires: menstrual pain, pain during sexual intercourse, cyclic pain during defecation, cyclic pain, infertility, and a high number of doctor/health care visits due to abdominal/pelvic pain. Recognising a broad range of potential symptoms is essential for raising awareness and supporting early detection efforts.

## 1 Introduction

Endometriosis is a chronic inflammatory condition characterised by a diverse and complex range of symptoms including abdominal and pelvic pain symptoms, gastrointestinal symptoms, fatigue and infertility [1, 2]. Symptoms can be constant, cyclical or non‐specific, mimicking other gynaecological, gastrointestinal and myofascial disorders [3, 4, 5, 6]. Endometriosis can be asymptomatic and may be circumstantially discovered while undergoing imaging or surgical procedures that visualise the pelvic cavity, e.g. in a fertility treatment work‐up or sterilisation [7, 8]. These factors, along with lack of awareness, menstrual stigma and missed diagnoses likely contribute to the significant diagnostic delay of 6–10 years [8, 9, 10, 11].

Based on prevalence estimates of pelvic pain and subfertility in the general population, endometriosis is estimated to affect 10% of people assigned female at birth of reproductive age [12]. However, the true prevalence of endometriosis is uncertain. Given that a definite diagnosis often involves laparoscopic visualisation, and there is a lack of pathognomonic symptoms, estimates of the population prevalence based on histologically verified diagnoses are likely to underestimate the true prevalence [1, 2, 13]. Furthermore, as diagnostic methods and definitions change over time, it impacts data upon which we can rely to calculate true prevalence in a population [14]. Numerous studies employing diverse study populations and methodologies report prevalence ranging from 1% to 18% [11, 13, 15, 16, 17, 18, 19, 20, 21, 22, 23, 24, 25, 26, 27]. Estimates derived from self‐reported data suggest a prevalence of 2%–8% [11, 15, 16, 17, 18, 19, 27]. Studies using diagnostic codes from hospitals and registries report prevalence between 1% and 18% [13, 21, 22, 23, 24, 25, 26]. Due to heterogenous population sampling, paths to referral for diagnosis, sampling scheme, endometriosis case definition and endometriosis documentation differences, these estimates vary significantly [14]. Many studies estimate the prevalence of endometriosis among highly selected populations such as at infertility centres, which cannot be generalised [14]. Thus, the true prevalence remains unknown.

To estimate the prevalence of endometriosis and related symptoms in a general population of individuals of reproductive age, and characterise patient populations, we are often restricted to questionnaire surveys. However, consensus on the most common symptoms and related consequences of endometriosis is lacking. Therefore, this study aimed to build consensus on symptoms and related consequences that can be used to characterise individuals with suspected and confirmed endometriosis in the general population. To achieve this objective, we have used a modified e‐Delphi method that involved patients with diagnosed endometriosis as well as experienced medical doctors and researchers working with endometriosis as experts.

## 2 Methods

We conducted a two‐round modified e‐Delphi questionnaire among an expert panel consisting of patients with diagnosed endometriosis, medical doctors and researchers working with endometriosis. The Delphi method consists of a series of controlled rounds, where repeated questionnaires are administered [28]. After each round of questionnaires, the participants are presented with an aggregated summary of the last round, allowing each expert to adjust their answers according to the group response. The Delphi method operates on the principle that predictions or decisions made by a structured group of individuals are more accurate than those from unstructured groups [29]. It has previously been used to develop medical recommendations, clinical guidelines, questionnaires, core outcomes and clinical indicators [30, 31]. The e‐Delphi method allows unbiased scoring by avoiding the influence of dominant individuals. Moreover, it encourages international participation and is generally regarded as a feasible, efficient and user‐friendly approach [28].

**FIGURE 1:**
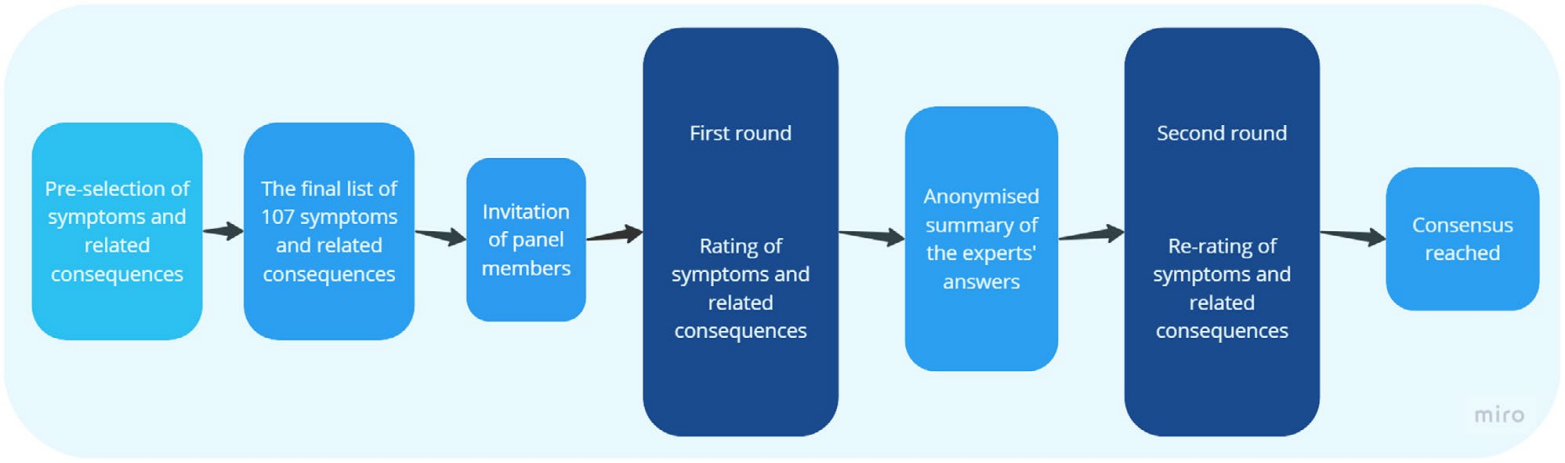
Illustration of the Delphi process in the current study.

In this study, we utilised this method to harness the insights of patients diagnosed with endometriosis, researcher and medical professionals specialising in the disorder. The participants answered the same set of questions in two rounds. After the first round, an anonymised summary of the participants answers from the previous round was provided. Participants were encouraged to revise their earlier answers based on the replies of the other participants. During this process, it was anticipated that the participants as a group would converge. The illustration of this Delphi process can be viewed in Figure 1.

The study was approved by the Danish Data Protection Agency under the Aarhus University comment agreement and Aarhus University j.number 2016–051‐000001, sequential number 2288 (Date: 7 April 2021).

### 2.1 Pre‐Selection of Symptoms and Related Consequences

A baseline questionnaire was developed with input from patients, researchers and medical doctors specialised in endometriosis. It encompasses both the suspected direct symptoms of endometriosis and the indirect consequences for affected women's lives. These consequences were deemed relevant to include, as they provide a more comprehensive understanding of the condition's impact.

Initially, a Facebook post was published inviting members of the Danish Endometriosis Society to share any symptoms and related consequences, they considered to be relevant for characterising individuals with suspected and confirmed endometriosis.

This list of symptoms and consequences was then sent to 11 experts, including medical doctors specialising in endometriosis, researchers in the field, and patients with the condition. They were requested to include any additional symptoms or consequences that they deemed relevant and not already present on the list. The final list comprised 107 symptoms suggestive of endometriosis and their related consequences.

The baseline questionnaire and invitation material were translated into English, Hungarian, Turkish and French by the collaborators who were native in the respective languages. We utilised the secure web application software research electronic data capture (REDCap) to build and manage online surveys facilitating the e‐Delphi process and data collection [32].

### 2.2 Panel Members

The panel consisted of individuals with endometriosis, medical doctors specialised in endometriosis, and endometriosis researchers from seven different countries (Australia, Denmark, France, Hungary, the United Kingdom, the United States, Turkey) to increase the cross‐cultural validity and usability. Recruitment took place through the FEMaLe project's network (EU Horizon2020 project titled “Finding Endometriosis using Machine Learning (FEMaLe)” (The FEMaLe project aiming to reduce diagnostic delay of endometriosis by utilising machine learning and artificial intelligence)), by the snowball sampling method, with collaborators from the project contributing to finding endometriosis related patients, medical doctors, and researchers in their network in their respective countries. Informed consent was obtained from all participants involved in this study. The inclusion criteria for the medical doctors and researchers were required to have experience working with endometriosis and the patients needed a self‐reported a priori diagnosis of endometriosis and a history of related symptoms to be included.

### 2.3 First Round

The first round was conducted from 17 November 2021 to 12 January 2022. Three reminders were sent with an interval of 2 days between each reminder. The initial round started with the dissemination of the first‐round questionnaire to all participants via email, with each email containing a private link for accessing the questionnaire. Initially, the participants were requested to provide their expert type, gender, age and geographical location. Subsequently, they were tasked with assessing the relevance of the 107 symptoms and related consequences listed in the baseline questionnaire by choosing an answer from a six‐point Likert scale with the following options: “not relevant”, “slightly relevant”, “relevant”, “very relevant”, “necessary”, or “unsure/outside my area of expertise” (Table A1). Moreover, participants could propose any number of additional relevant symptoms and related consequences that they considered appropriate for the second round.

### 2.4 Second Round

The second round was conducted between 15 February 2022 and 6 April 2022. Two reminders were sent with an interval of 3 days between each reminder. Each of the participants who had participated in the first round, was sent the second‐round questionnaire by e‐mail, accompanied by a personal link. In round two, participants were sent the summary statistics from Round 1 including the graphical distribution of answers of each symptom. The participants were asked to re‐rate the selected symptoms and related consequences based on their own opinion and the panels responses obtained during the first round.

### 2.5 Data Analysis

It was established a priori that a symptom would be considered relevant for characterising individuals with endometriosis if at least 70% of the participants rated it as “*very relevant*” or “*necessary*” [33]. Further, to ensure equal weighting between the input of patients and medical doctors/researchers, the percentage ratings from both groups were added and then divided by two.

The distribution of answers for each symptom was stratified between patients and medical doctors/researchers to identify any potential differences that might exist between the two groups.

In compliance with General Data Protection Regulation (GDPR), we limited the display of data to ensure participant privacy. Therefore, groups with fewer than five individuals were not revealed. This restriction is the reason the numbers in the studies Tables 2 and A1 must be between 20% and 80%.

## 3 Results

A total of 79 participants were invited to join the modified e‐Delphi expert panel, comprising 36 medical doctors/researchers and 43 patients. Of these, 76 (96%) successfully completed the first round of the questionnaire (33 medical doctors/researchers and 43 patients). Thirteen males and 63 females participated. The participants' age ranged between 22 and 69. Half of the participants were below 39 years of age. In terms of geographic location, the highest percentage of participants were from English speaking countries, such as Australia, the United Kingdom and the United States. These were followed by Turkey, Denmark, Hungary and France, ranked in descending order (Table 1).

**TABLE 1:** Participant characteristics.

	Round 1 *n* = 76	Round 2 *n* = 65
Participants, *n* (%)		
Medical doctors and researchers	33 (43%)	27 (42%)
Patients	43 (57%)	38 (58%)
Gender, *n* (%)		
Male	13 (17%)	11 (17%)
Female	63 (83%)	54 (83%)
Age (years), *n* (%)		
Under 39	38 (50%)	35 (54%)
40–49	24 (32%)	20 (31%)
Above 50	14 (18%)	10 (15%)
Geographical location, *n* (%)		
Australia, the United Kingdom, and the United States	27 (36%)	23 (35%)
Denmark	13 (17%)	9 (14%)
France	7 (9%)	5 (8%)
Hungary	11 (14%)	11 (17%)
Turkey	18 (24%)	17 (26%)

Among the 107 symptoms and related consequences in the first round, four symptoms met the cut‐off of being considered very relevant or necessary to endometriosis by 70% or more of the participants. These were menstrual pain (> 80%), pain during sexual intercourse (75%), cyclic pain during defecation (73%), and infertility (71%) (Table 2).

**TABLE 2:** Symptoms and related consequences which achieved consensus after Round 2.

	Round 1 *n* = 76	Round 2 *n* = 65
Symptoms		
Menstrual pain	> 80%	> 80%
Pain during sexual intercourse	75%	79%
Cyclic pain during defecation	73%	70%
Cyclic pain	69%	79%
Infertility	71%	79%
A high number of doctor/health care visits due to abdominal/pelvic pain	63%	73%

Ten additional symptoms and related consequences were added to the baseline questionnaire in the second round (Table A1). In this round, 65 participated (27 medical doctors/researchers and 38 patients). Out of a total of 117 symptoms, six symptoms and related consequences reached consensus, including the four from the first round: menstrual pain (> 80%), pain during sexual intercourse (79%), cyclic pain during defecation (70%), and infertility (79%), along with cyclic pain (79%), and a high number of doctor/hospital contacts due to pelvic pain (73%) (Table 2).

In terms of differences between medical doctors/researchers and patients, it appears that medical doctors tend to favour the prognostic indicators, such as *pain and a high level of absence from work/school*, while patients prioritise symptom‐related feelings such as *feeling that your health is outside of your control* and *feeling frustrated because your symptoms are not getting better* (Table A1). Consensus was reached on four symptoms and related consequences by only one group, either medical doctors/researchers or patients. These symptoms and related consequences include *pelvic pain between menstruations*, with patients reaching consensus at 74% compared to medical doctors/researchers at 59%. Additionally, for *a high level of absence from work/school*, medical doctors/researchers reached consensus at 70%, while patients only reached 34%. For *avoiding sexual intercourse*, medical doctors/researchers reached consensus at 78%, whereas patients reached 42%. Moreover, *heavy menstrual bleeding*, reached consensus among the patients at 71%, compared to the medical doctors/researchers at 33% (Table A1).

## 4 Discussion

### 4.1 Main Findings

In this study, we used a modified e‐Delphi method to gather insights on symptoms and related consequences that may be relevant for inclusion in a questionnaire to help characterise individuals with suspected and confirmed endometriosis in the general population. To accomplish this, a panel of experts consisting of individuals with endometriosis and medical doctors and researchers, who were experts in endometriosis, evaluated the relevance of 117 predefined symptoms. Six symptoms and related consequences reached consensus: *menstrual pain, pain during sexual intercourse, cyclic pain during defecation, cyclic pain, infertilit*, and *a high number of doctor/health care visits due to abdominal/pelvic pain*.

### 4.2 Strengths and Limitations

The strength of this study lies in its multinational and multicultural design, providing a broad and diverse perspective on the subject. Including participants from different countries and cultural backgrounds enhances the generalisability of findings, acknowledging and accounting for potential variations in experiences, perspectives and healthcare practices across diverse populations [34]. This approach strengthens the study's external validity [34]. However, a limitation is the uneven distribution of patients, medical doctors and researchers across the participating countries. This may impact the representativeness of perspectives, potentially reducing the generalisability of our findings [34]. Increasing the sample size would contribute to the reliability and generalisability of our findings, enhancing the validity of the consensus reached in subsequent rounds of the e‐Delphi process [34]. Furthermore, a larger sample size would allow for examining potential differences between countries, adding additional valuable insights to our study.

Another strength of this study is the involvement of both patients and medical doctors in the selection of the relevant symptoms and related consequences. The Delphi process, mixing different stakeholders, made it possible to select elements that are easy to understand and represent the subjective experience of endometriosis, but that are also useful from a medical point of view [35]. Additionally, the Delphi method has the advantage over other consensus methods that individuals can be included anonymously and without interacting directly with each other, which prevents the views of a minority from dominating the group [34, 36]. Another notable strength is the low occurrence of individuals discontinuing their involvement, contributing to increased validity and consequently, allowing for a more consistent and reliable consensus‐building process [37].

Finally, a limitation arises from the requirement that a symptom is deemed relevant if at least 70% of participants rate it as “*very relevant*” or “*necessary*”. This limits symptoms to those considered indicative of endometriosis based on current definitions and diagnostic practices. It also ignores symptoms that may be highly relevant to as yet unknown subtypes of endometriosis. Adjusting the consensus threshold percentage may broaden the spectrum of symptoms leading to more women with suspected endometriosis to be identified. It is, however, important to strike a balance between the objective of identifying those with endometriosis and maintaining a manageable questionnaire length to ensure optimal response rates [38]. Furthermore, it is essential to recognise that although many symptoms did not gain consensus, they should not be disregarded as they were considered significant by some participants.

### 4.3 Interpretation

The symptoms and related consequences found in this study have previously been found to be common in individuals with endometriosis [7, 16, 27, 39, 40, 41, 42]. These can, however, also be associated with other conditions [42, 43] and are therefore not unique to those with endometriosis. Further, they are often symptoms necessary to triage a patient for imaging or certainly surgical evaluation at which endometriosis—particularly the most common superficial peritoneal subtype presentation—can be diagnosed. A previous study has estimated the sensitivity and specificity of self‐reported severe dysmenorrhea, pain during sexual intercourse, chronic pelvic pain and infertility for identifying individuals with endometriosis confirmed by surgery [43]. For all symptoms the specificity was found to be relatively high (70%–96%) but the sensitivities were generally low (16%–58%) [43]. The low sensitivity suggests that relying on these specific symptoms alone for identifying endometriosis may result in a considerable number of individuals with endometriosis being overlooked. Also, assuming a true prevalence of endometriosis at 10% or below, the predictive value of a positive test based on these symptoms would still be low, despite the relatively high specificities for the suggested symptoms.

Previous research indicates that the presence of a high number of symptoms increases the likelihood of referral for a procedure at which endometriosis can be diagnosed. This is evident when considering symptoms collectively, including dysmenorrhea, abdominopelvic pain, symptoms associated with sexual intercourse, urinary tract symptoms and fertility issues [39]. The odds ratios for predicting endometriosis indicate a progression, starting at 5 (95% CI: 4.4–5.7) for one symptom and reaching 84.7 (95% CI: 58.8–121.8) for seven or more symptoms [39]. The study suggests that the presence of a symptom cluster may be important for predicting endometriosis in individuals [39]. While these symptom clusters may help confirm the presence of endometriosis in those who test positive, this approach most likely comes at the cost of overlooking others with endometriosis. More research is needed to adjust the balance of sensitivity, specificity and predictive value of symptoms related to endometriosis, considering that there is no true gold standard against which to calculate accurate sensitivity, specificity and predictive values. The selection of symptoms should reflect the purpose of the task. If the aim is to identify individuals who, with a high degree of certainty, have endometriosis, then symptom clusters most likely are the relevant choice. However, attempting to estimate the true prevalence of endometriosis solely based on symptoms might be insufficient, as it may lead to underdiagnosis or overdiagnosis depending on the selected population. It is important to acknowledge that recognising a wide range of potential symptoms associated with endometriosis remains crucial for raising awareness and promoting early detection. Despite their limitations in diagnostic accuracy, symptoms serve as valuable indicators for prompting further evaluation and assessment.

Interesting differences in the types of symptoms valued by the different groups of participants were found. It was observed that medical doctors might be more likely to prioritise items with a prognostic value for patient management, indicating their preference for signs that contribute to effective medical disease management. Patients, however, appear to be more likely to express their discomfort and emotional distress due to pain and changes to their quality of life [44].

As for future perspectives of this consensus study, the results have been incorporated into the Danish CYKLUS questionnaire, which was sent to a random sample of approximately 63 000 Danish individuals in the spring of 2023 [45]. The questionnaire aims to contribute to knowledge about general health, symptoms, diseases, and well‐being for individuals, who were born female. Further, we aim to estimate the prevalence of the six endometriosis related symptoms and consequences found in this study to see the distribution of these symptoms in Denmark among individuals of reproductive age.

## 5 Conclusion

This consensus study highlighted six symptoms and related consequences commonly associated with endometriosis: menstrual pain, pain during sexual intercourse, cyclic pain during defecation, cyclic pain, infertility, and frequent doctor/health care visits for abdominal/pelvic pain. Recognising a broad range of potential symptoms is essential for raising awareness and supporting early detection efforts, and these symptoms may be useful for inclusion in future questionnaires aimed at characterising individuals with suspected and confirmed endometriosis among the general population.

## Author Contributions

T.Z. was responsible for data curation, visualisation, original draft writing and review and editing. H.M. contributed to data curation writing review and editing. K.E.H. managed conceptualization, methodology, supervision and writing review and editing. A.W.H., L.S., K.T.Z., S.A.M. and L.H. was responsible for recruitment, writing review and editing. A.B. assisted with recruitment. A.M. and C.S.Ø. contributed to writing review and editing. D.R. was responsible for conceptualization, methodology, project administration, recruitment, supervision, validation and writing review and editing. All authors accept responsibility for the paper as published.

## Ethics Statement

The study was approved by the Danish Data Protection Agency under the Aarhus University comment agreement and Aarhus University journal number 2016‐051‐000001, sequential number 2288 (Date: 7 April 2021). All participants gave full consent for this specific study.

## Conflicts of Interest

The authors declare no conflicts of interest.

## Supporting information


Table A1:

## Data Availability

Research data are not shared.
