# Supplementary material for: Consensus on Symptom Selection for Endometriosis Questionnaires: A Modified e‐Delphi Study
Source: BJOG. 2025 Jan 13;132(5):656–62. doi: 10.1111/1471-0528.18066 (PMC11879915; doi:10.1111/1471-0528.18066)
Supplement: Supplementary file 1 — Table A1: [file BJO-132-656-s001.docx]

**APPENDIX**

Appendix table 1. Proportion of patients and doctors/researchers assessing symptoms very relevant or necessary to detect women with undiagnosed or diagnosed endometriosis (Sorted “All” from highest to lowest).

| Nr. | Symptoms | Patients who responded with, “very relevant” and “necessary” (%) | Medical doctors/ researchers who responded with, “very relevant” and “necessary” (%) | All * (%) |
| --- | --- | --- | --- | --- |
| 1 | Menstrual pain | >80 | >80 | >80 |
| 3 | Pain during sexual intercourse | 68 | >80 | 79 |
| 30 | Cyclic pain | 76 | >80 | 79 |
| 38 | Infertility / difficulty getting pregnant | 74 | >80 | 79 |
| 86 | A high number of doctor / health care visits due to abdominal / pelvic pain | 71 | 74 | 73 |
| 5 | Cyclic pain during defecation | 63 | 78 | 70 |
| 2 | Pelvic pain in between menstruations | 74 | 59 | 66 |
| 72 | Feeling unable to do the things you want because of the pain | 68 | 59 | 64 |
| 103 | Avoiding sexual intercourse ** | 42 | 78 | 60 |
| extra6 | Pain before menstruation | 63 | 56 | 59 |
| 28 | Cyclic pain radiating to the groin | 55 | 59 | 57 |
| 77 | Feeling unable to cope with the pain | 61 | 52 | 56 |
| extra10 | Endometriosis in close relatives | 55 | 52 | 54 |
| 6 | Cyclic pain during urination | 42 | 63 | 53 |
| extra3 | Pain in the presumed area of the ovaries | 58 | 48 | 53 |
| extra5 | Absence from work/school due to pain | 47 | 59 | 53 |
| 91 | A high level of absence from work / school | 34 | 70 | 52 |
| extra9 | Heavy menstrual bleeding ** | 71 | 33 | 52 |
| 40 | Heavy menstrual bleeding ** | 66 | 37 | 51 |
| 95 | Worrying about not being able to carry out your job / schoolwork | 45 | 52 | 48 |
| 102 | Worrying about having sexual intercourse | 45 | 52 | 48 |
| 73 | Feeling that your health is outside of your control | 61 | 33 | 47 |
| 75 | Feeling frustrated because your symptoms are not getting better | 61 | 33 | 47 |
| 76 | Feeling frustrated because you are not able to control your symptoms | 53 | 41 | 47 |
| 27 | Cyclic pain radiating to the legs | 42 | 48 | 45 |
| 74 | Feeling that the symptoms are taking away your life | 55 | 33 | 44 |
| 81 | Worrying about pain | 50 | 37 | 44 |
| 92 | Inability to carry out tasks at work / school | 32 | 56 | 44 |
| 104 | Feeling guilty about not wanting to have sexual intercourse | 39 | 48 | 44 |
| 4 | Pain after sexual intercourse | 45 | 41 | 43 |
| 89 | Frustration about the doctor's / health care provider's lack of knowledge about endometriosis | 53 | 33 | 43 |
| 105 | Frustration because you cannot enjoy sexual intercourse | 26 | 59 | 43 |
| 47 | Fatigue / lack of energy / exhaustion | 58 | 26 | 42 |
| 94 | Feeling guilty about taking time off work / school | 37 | 48 | 42 |
| extra8 | Use of opiates for abdominopelvic pain | 39 | 44 | 42 |
| 29 | Cyclic chest or shoulder pain | 34 | 48 | 41 |
| 96 | Difficulty participating in social events | 47 | 33 | 40 |
| 15 | Feeling bloated | 58 | <20 | 38 |
| 26 | Cyclic pain radiating to the lower and upper back | 32 | 44 | 38 |
| 57 | A feeling that others do not understand what you are going through | 50 | 26 | 38 |
| 78 | Feeling unable to forget the symptoms | 47 | 30 | 38 |
| 101 | Difficulty performing physical activity / exercise | 47 | 26 | 37 |
| 79 | Stress | 45 | 26 | 35 |
| 82 | Worrying about the future | 45 | 26 | 35 |
| 87 | A feeling that your doctor(s) / health care provider(s) are not doing anything for you | 34 | 33 | 34 |
| 93 | Feeling embarrassed about symptoms at work / school | 29 | 37 | 33 |
| 39 | Irregular bleeding | 45 | <20 | 32 |
| 58 | A feeling that others think you are moaning / complaining | 42 | 22 | 32 |
| 88 | A feeling that the doctor(s) / health care provider(s) think that it is all in your mind | 32 | 33 | 32 |
| 99 | Difficulty managing daily chores / activities at home (including cleaning, shopping, laundry, bathing etc.) | 24 | 37 | 30 |
| 7 | Pain at ovulation | 37 | <20 | 28 |
| extra7 | Difficulty holding down a job/career | 26 | 30 | 28 |
| 25 | Lower back pain | 47 | <20 | 27 |
| 16 | A feeling of heaviness in the abdomen | 45 | <20 | 26 |
| 60 | A feeling of helplessness | 34 | <20 | 26 |
| 106 | Difficulties looking after your child / children | <20 | 33 | 26 |
| 10 | Rectum cramps / spasms | 42 | <20 | 25 |
| 61 | A feeling of not being sufficient | 32 | <20 | 25 |
| 65 | Mood swings | 39 | <20 | 25 |
| 8 | Stomachache | 37 | <20 | 24 |
| 83 | Feeling deflated about the future | 29 | <20 | 24 |
| 100 | Difficulty finding capacity to enjoy hobbies | 26 | 22 | 24 |
| extra4 | Pain when running or cycling | 37 | <20 | 24 |
| 14 | Constipation | 34 | <20 | 23 |
| 80 | Feeling anxious / suffering from anxiety | 32 | <20 | 23 |
| 84 | Feeling mistrusted due to symptoms | 34 | <20 | 23 |
| extra2 | Pain after orgasm | 26 | <20 | 22 |
| 11 | Blood and mucus in feces | 34 | <20 | 21 |
| 64 | A changed view of yourself | 26 | <20 | 21 |
| 63 | Low self-esteem | 29 | <20 | 20 |
| 66 | Feeling bad tempered or short tempered | 29 | <20 | 20 |
| 9 | Pain after having eaten | <20 | <20 | <20 |
| 12 | Blood in urine | <20 | <20 | <20 |
| 13 | Diarrhea | 24 | <20 | <20 |
| 17 | Frequent urge to use the toilet (to open bowels or pass urine) | 26 | <20 | <20 |
| 18 | Acute urge to use the toilet (to open bowels or pass urine) | 29 | <20 | <20 |
| 19 | Lack of appetite | <20 | <20 | <20 |
| 20 | Food allergies | <20 | <20 | <20 |
| 21 | Joint pain | <20 | <20 | <20 |
| 22 | Muscle pain | <20 | <20 | <20 |
| 23 | Headache / migraine | <20 | <20 | <20 |
| 24 | Tensions in the jaw | <20 | <20 | <20 |
| 31 | Pain when standing | 24 | <20 | <20 |
| 32 | Pain when sitting | 21 | <20 | <20 |
| 33 | Pain when walking | <20 | <20 | <20 |
| 34 | Pain when lying down | <20 | <20 | <20 |
| 35 | Pain during physical activity | 26 | <20 | <20 |
| 36 | Pain sensitivity to cold | <20 | <20 | <20 |
| 37 | Pain sensitivity to tight clothing | 29 | <20 | <20 |
| 41 | 'Flu-like' / 'fever-like' symptoms | <20 | <20 | <20 |
| 42 | Generally feeling unwell | 24 | <20 | <20 |
| 43 | Nausea | <20 | <20 | <20 |
| 44 | Vomiting | <20 | <20 | <20 |
| 45 | Urinary tract infection / cystitis | <20 | <20 | <20 |
| 46 | Sleeping poorly / insomnia | 21 | <20 | <20 |
| 48 | Impaired memory | <20 | <20 | <20 |
| 49 | Impaired ability to concentrate | <20 | <20 | <20 |
| 50 | A general lack of perspective | <20 | <20 | <20 |
| 51 | Impaired ability to plan | <20 | <20 | <20 |
| 52 | Rapid heartbeat | <20 | <20 | <20 |
| 53 | Dizziness | <20 | <20 | <20 |
| 54 | Breathing problems | <20 | <20 | <20 |
| 55 | Pain when breathing | <20 | <20 | <20 |
| 56 | A feeling of loneliness | <20 | <20 | <20 |
| 59 | A feeling of insecurity | 26 | <20 | <20 |
| 62 | A feeling of being worthless | 24 | <20 | <20 |
| 67 | Anger | <20 | <20 | <20 |
| 68 | Feeling violent or aggressive | <20 | <20 | <20 |
| 69 | Feeling depressed | 21 | <20 | <20 |
| 70 | Feeling weepy or tearful | <20 | <20 | <20 |
| 71 | Feeling miserable | 24 | <20 | <20 |
| 85 | Feeling dejected | 21 | <20 | <20 |
| 90 | A feeling like you are wasting the doctor's / health care provider's time | 21 | <20 | <20 |
| 97 | Difficulty participating in a conversation | <20 | <20 | <20 |
| 98 | Difficulty participating in large gatherings | 21 | <20 | <20 |
| 107 | Inability to play with your child / children | <20 | 26 | <20 |
| extra1 | Bleeding after sexual intercourse | 21 | <20 | <20 |

* The average of responses from patients and medical doctors/researchers.
** The questionnaire included heavy menstrual bleeding on two occasions.
